# Feeding bovine milks with low or high IgA levels is associated with altered re-establishment of murine intestinal microbiota after antibiotic treatment

**DOI:** 10.7717/peerj.2518

**Published:** 2016-09-29

**Authors:** Alison J. Hodgkinson, Wayne Young, Julie A. Cakebread, Brendan J. Haigh

**Affiliations:** 1 Food & Bio-based Products, AgResearch, Hamilton, New Zealand; 2 Food & Bio-based Products, AgResearch, Palmerston North, New Zealand

**Keywords:** Immunoglobulin A, Milk, Microbiota, Intestine, Antibiotics

## Abstract

Antibiotics are a vital and commonly used therapeutic tool, but their use also results in profound changes in the intestinal microbiota that can, in turn, have significant health consequences. Understanding how the microbiota recovers after antibiotic treatment will help to devise strategies for mitigating the adverse effects of antibiotics. Using a mouse model, we have characterized the changes occurring in the intestinal microbiota immediately after five days exposure to ampicillin, and then at three and fourteen days thereafter. During the fourteen day period of antibiotic recovery, groups of mice were fed either water, cows’ milk containing high levels of IgA, or cows’ milk containing low levels of IgA as their sole source of liquid. Effects on microbiota of feeding milks for 14 days were also assessed in groups of mice that had no ampicillin exposure. Changes in microbiota were measured by high throughput sequencing of the V4 to V6 variable regions of the 16S ribosomal RNA gene.

As expected, exposure to ampicillin led to profound changes to the types and abundance of bacteria present, along with a loss of diversity. At 14 days following antibiotic exposure, mice fed water had recovered microbiota compositions similar to that prior to antibiotics. However, feeding High-IgA milk to mice that has been exposed to antibiotics was associated with altered microbiota compositions, including increased relative abundance of *Lactobacillus* and *Barnesiella* compared to the start of the study. Mice exposed to antibiotics then fed Low-IgA milk also showed increased *Barnesiella* at day 14. Mice without antibiotic perturbation, showed no change in their microbiota after 14 days of milk feeding. Overall, these findings add to a knowledge platform for optimizing intestinal function after treatment with antibiotics in the human population.

## Introduction

Antibiotics are administered widely in the human population (Col & O’Connor, 1987) and are a vital therapeutic tool for combating infection. However, the presence of antibiotics within the digestive tract leads to large scale alteration of the intestinal microbiota (Fouhy et al., 2012; Jakobsson et al., 2010; Mikkelsen et al., 2015a). Paradoxically, this has been shown to increase susceptibility to further pathogenic infection by making available niches previously occupied by commensal bacteria, or by reducing competition for resources (Ng et al., 2013; Schubert, Sinani & Schloss, 2015). The use of antibiotics can also lead to the persistence of antibiotic-resistant strains of bacteria (Ubeda et al., 2010).

Recent published findings have underscored the importance of an optimal balance of microbes in the intestine for a range of functions, including energy metabolism (DiBaise, Frank & Mathur, 2012) and immune function (Chung et al., 2012; Hooper, Littman & Macpherson, 2012). The makeup of the intestinal microbiota has been shown to shown to influence conditions such as obesity (Azad et al., 2014; Schwartz et al., 2016), Type-2 diabetes (Mikkelsen et al., 2015b), allergic disease (McCoy & Koller, 2015), as well as mental health (Cryan & Dinan, 2012). Changes to microbiota by the administration of antibiotics may, therefore, result in significant health consequences. Currently there is no consensus for optimal management practises to minimise impact of antibiotic usage on the patient.

The selection of food consumed is generally considered to influence the types and prevalence of microbes present in the intestine (Saarela et al., 2002). Ingestion of fermented foods and cultured products containing probiotic bacteria, such as *Lactobacillus* and *Bifidobacterium*, are considered to be beneficial for gut health, although, as yet there is only limited evidence for significant health benefits of this practise (Di Cerbo et al., 2016; Martinez, Bedani & Saad, 2015). An alternative approach is ingestion of prebiotics; defined as a non-viable food component that confers a health benefit on the host and that is associated with modulation of microbiota (Roberfroid et al., 2010). The role of prebiotics in gut health have being illustrated in a number of studies, e.g., resistant starches (Bindels, Walter & Ramer-Tait, 2015) and human milk oligosaccharides (Bode, 2015).

The composition of milk has been optimised through the evolution of mammals to provide the sole support for growth and development of suckling offspring. In addition to proteins, fats and carbohydrates, milk also contains a range of immunomodulatory components, including immunoglobulin A (IgA). Milk-derived IgA plays an important role in the optimisation, establishment and maintenance of the microbial milieu within the intestinal lumen of the neonate (reviewed in Cakebread, Humphrey & Hodgkinson (2015)). IgA is a heavily glycated protein (Froehlich et al., 2010; Imperiali & O’Connor, 1999; Mathias & Corthesy, 2011), that is protected from proteolysis by secretory component (Crottet & Corthesy, 1998; Lindh, 1975) and has both immune inclusion and immune exclusion properties (Cakebread, Humphrey & Hodgkinson, 2015).

In this study, we tested the hypothesis that ingestion of milk facilitates the recovery of the microbial intestinal populations after antibiotic exposure, and this is mediated in part by milk-IgA. Using a mouse model, we have characterised the changes occurring in the intestinal microbiota immediately after five days exposure to ampicillin, and then at three and fourteen days thereafter. During the fourteen day period of antibiotic recovery, groups of mice were fed either water, cows’ milk containing high levels of IgA, or cows’ milk containing low levels of IgA as their sole source of liquid. Effects on microbiota of feeding milks for 14 days were also assessed in groups of mice that had no ampicillin exposure. Temporal changes in microbiota over the study period, measured by high throughput sequencing of the V4 to V6 variable regions of the 16S ribosomal RNA gene, are reported here.

## Materials and Methods

### Milk collection and IgA measurement

Milk was obtained from pasture grazed Jersey-Friesian cows that were part of a commercial milking herd. The cows were at mid-lactation and milked on a twice-a-day regimen. Levels of IgA in their milks had previously been measured by ELISA using a commercially supplied kit (Bethyl Laboratories, Montgomery, TX, USA), according to the manufacturer’s recommendations. Milks were collected at a single milking. Two separate pools were created by mixing equal volumes of milk from three cows with high levels of IgA and three cows with low levels of IgA. The IgA concentrations in the milk pools were measured by ELISA; the concentration of IgA in the High-IgA and Low-IgA milk were 0.73 mg/ml and 0.09 mg/ml, respectively. The milks were stored frozen at −20 °C until used in the mouse-feeding experiment. Milk composition information is listed in Table S1.

### Housing and treatment of mice

Animal experiments were performed in accordance with the guidelines of the New Zealand National Animal Ethics Advisory Committee for the use of animals in research, testing and teaching and approved by the Ruakura Animal Ethics Committee (AEC#13356). A total of 60 Balb/C mice, aged between 10 and 14 weeks, were divided into two sets of three treatment groups, each group comprising five males and five females. Groups were housed in 2 cages containing five mice of a single sex, per treatment. All mice were offered standard mouse chow (dairy free) *ad libitum* throughout the experimental period. Mice were weighed weekly and monitored daily for signs of ill health or discomfort. Set 1 (groups 1–3) were not exposed to antibiotics and offered water (group 1), High-IgA milk (group 2) or Low-IgA milk (group 3) for 14 days. Set 2 (groups 4–6) were exposed to 1 mg/ml ampicillin in their drinking water for five days, then offered water (group 4), High-IgA milk (group 5) or Low-IgA milk (group 6) for 14 days. Each treatment was delivered via a sipper bottle as the only source of liquid intake. Fluid intake was monitored by weighing each bottle daily before replenishing it with fresh water or milk. At various time points, faecal pellets were collected by placing each mouse in an individual container until it had passed two to three pellets. For groups not exposed to ampicillin (groups 1–3), a pre sample of faecal pellets was collected before water/milk feeding, then a second sample was collected at day 14 of the water/milk treatment period. For ampicillin-exposed mice, faecal pellets were collected prior to exposure to ampicillin, then after ampicillin exposure at day 0, at day 3 and at day 14 of the water/milk treatment period. The pellets were stored at −20 °C until analysed.

### 16S ribosomal RNA analysis

The faecal pellets from each mouse were thawed and homogenised in PBS to achieve a suspension of 100 mg pellet per ml. Bacterial DNA was extracted from the faecal homogenate using NucleoSpin Soil kits (Macherey Nagel, Düren, Germany). Microbiota profiling was assessed by barcode pyrosequencing of bacterial 16S rRNA gene PCR products, as described previously (Young et al., 2015). Purified PCR products were pooled in equimolar amounts and sent to Macrogen (Seoul, Korea) for sequencing using the GS-FLX Titanium System (Roche). Sequences were processed using the Qiime 1.8 pipeline (Caporaso et al., 2010) with default quality filtering parameters followed by chimera removal using the USEARCH method. Sequences were clustered into operational taxonomic units (OTUs) using the UCLUST method (0.97 similarity) and representative sequences were assigned taxonomies using the RDP classifier with an 80% confidence threshold. Differences between communities were visualised using Principal Coordinate Analysis (PCoA) of weighted Unifrac phylogenetic distances. Differences in diversity was assessed using Faith’s Phylogenetic Diversity in Qiime.

### Statistical analysis

Statistical analyses were performed using R 3.1.3 (R Development Core Team, 2011). Differences between mean relative abundance of individual taxa among the different treatments at day 3 and day 14 were assessed for significance using the Kruskal–Wallis analysis of variance in *R*. Kruskal-Wallis *P* values for analyses below the phylum level were adjusted for multiple testing using the Benjamini Hochberg false discovery rate (FDR) method. Changes in taxa over time for each group, with or without antibiotic exposure, was also assessed using paired Wilcoxon rank sum test. Taxa with an FDR <0.05 were considered significantly different.

## Results

### Comparison of overall community structure without antibiotic exposure

**Figure 1:**
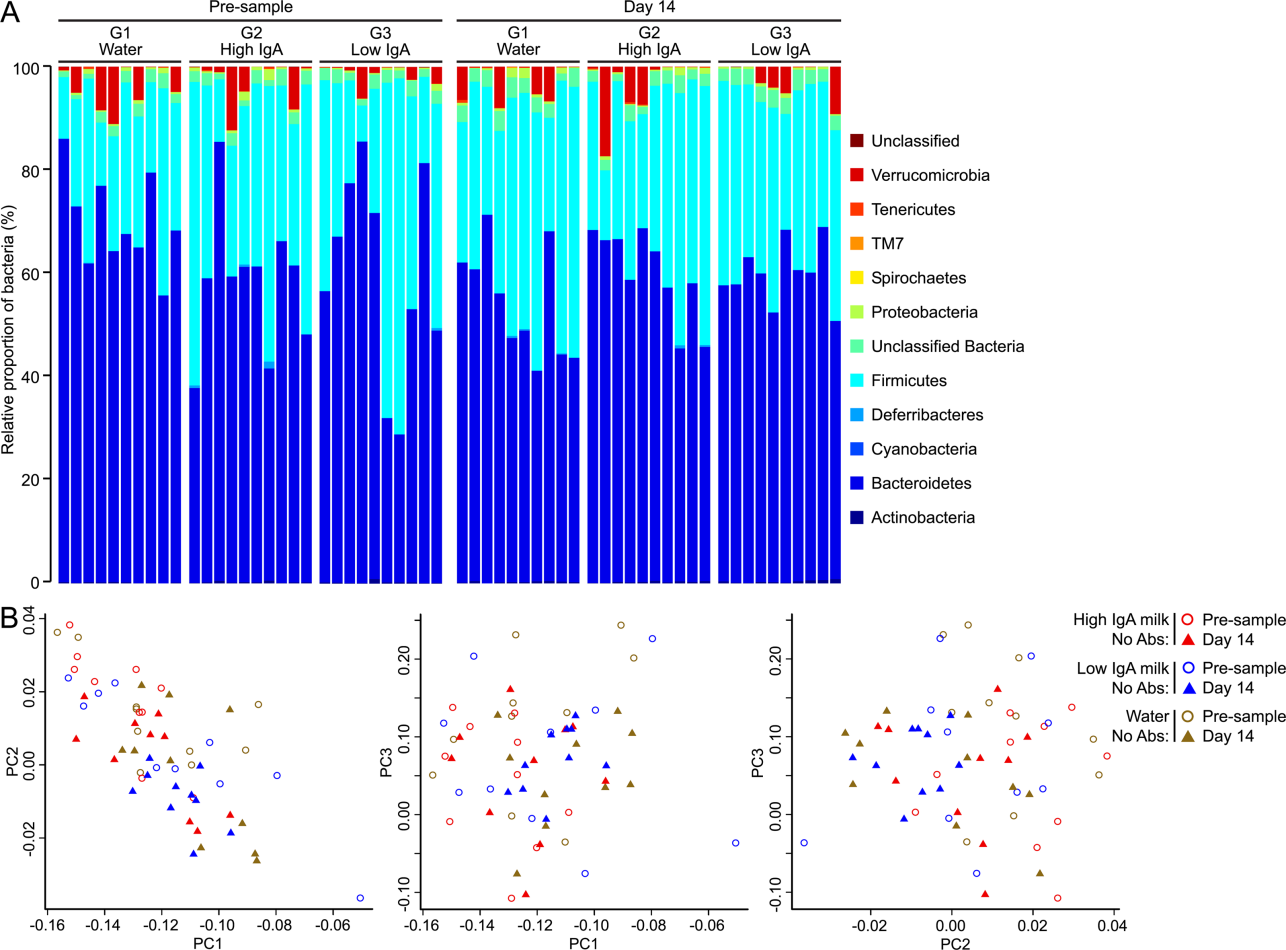
Changes to bacterial communities without antibiotic exposure. (A) The relative abundance of phyla present in faecal pellets collected from individual mice that received no exposure to antibiotics and fed water/milk for a period of 14 days are shown for Group 1 (water), Group 2 (High-IgA milk) and Group 3 (Low-IgA milk) at the beginning of the experiment (Pre) and at day 14 of water/milk treatment. The colours represent different phyla as indicated in the figure legend. (B) Principle Co-ordinate plots for 16S rDNA sequencing data of bacterial communities (PC1 versus PC2, PC1 versus PC3 and PC2 versus PC3) in individual mice that received no exposure to antibiotics and fed water/milk for a period of 14 days are shown for Group 1 (water, brown symbols), Group 2 (High-IgA milk, red symbols) and Group 3 (Low-IgA milk, blue symbols) at the beginning of the experiment (Pre, open circles) and after 14 days treatment (Day 14, solid triangles).

**Figure 2:**
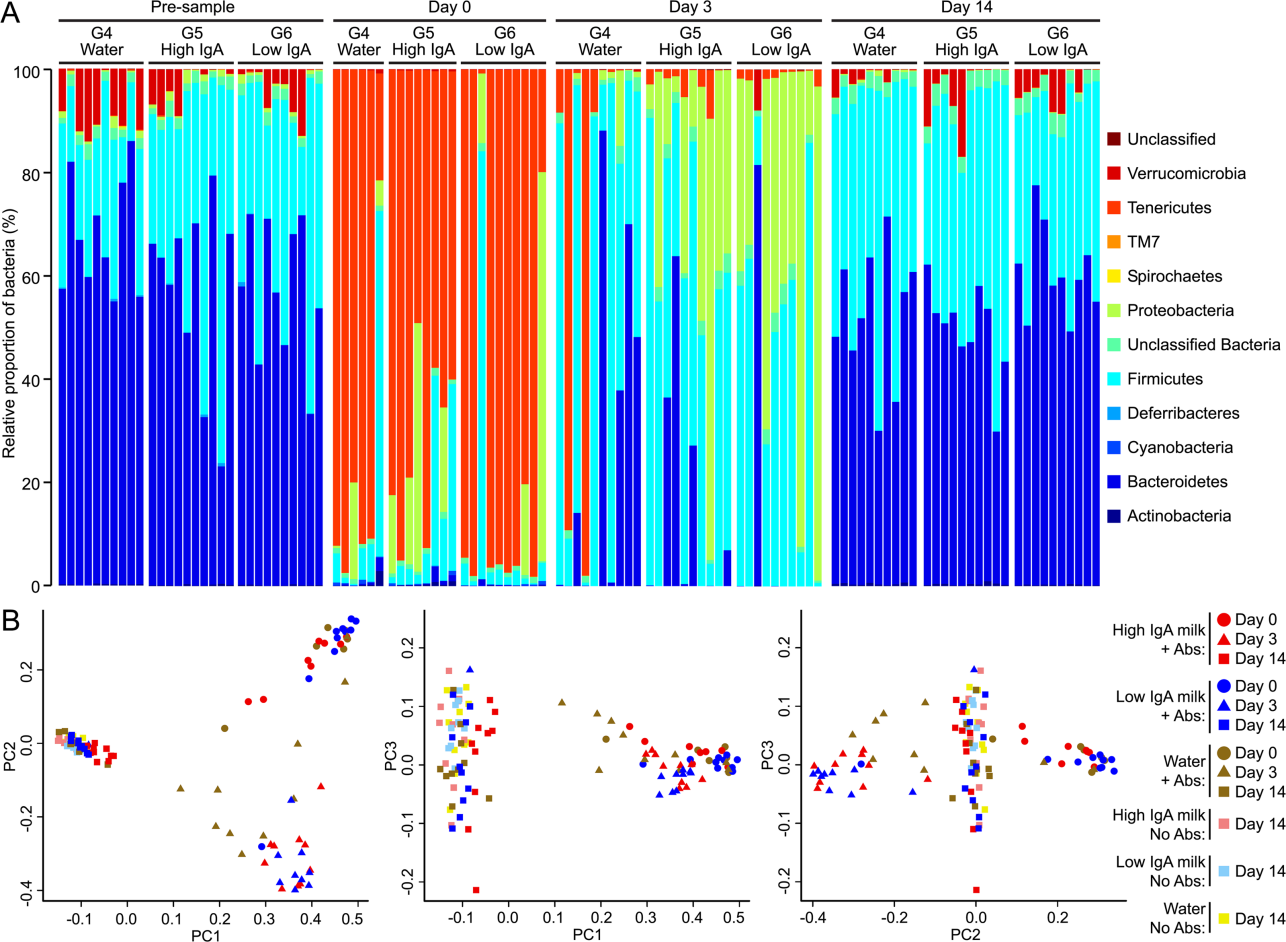
Changes to bacterial communities following antibiotic exposure. (A) The relative abundance of phyla present in faecal pellets collected from individual mice that were exposed to antibiotics and then fed water/milk for a period of 14 days are shown for Group 4 (water), Group 5 (High-IgA milk) and Group 6 (Low-IgA milk) at the beginning of the experiment (Pre), immediately after five days of antibiotic exposure at day 0, then at day 3 and day 14 of the water/milk treatment period. The colours represent different phyla as indicated in the figure legend. (B) Principle Co-ordinate plots for 16S rDNA sequencing data of bacterial communities (PC1 versus PC2, PC1 versus PC3 and PC2 versus PC3) in individual mice that were exposed to antibiotics and then fed water/milk for a period of 14 days are shown for Group 4 (water, brown symbols), Group 5 (High-IgA milk, red symbols) and Group 6 (Low-IgA milk, blue symbols) at Day 0 (solid circles), Day 3 (solid triangles) and Day 14 (solid triangles). For comparison, Day 14 data for individual mice that received no exposure to antibiotics and fed water/milk for a period of 14 days are also shown; Group 1 (water, yellow solid squares), Group 2 (High-IgA milk, light red solid squares) and Group 3 (Low-IgA milk, light blue solid squares).

Prior to any treatments, the faecal microbiota in all groups consisted primarily of the phyla *Bacteriodetes* and *Firmicutes*, accounting for 60% and 35% of the communities, respectively (Figs. 1A & 2A, Pre). No significant differences were observed between the faecal microbiota of male and female mice. In groups of mice that received no antibiotic exposure, a 14 day feeding period of milk (either Low- or High-IgA milk) made no significant change to the microbiota communities (Fig. 1A, Day 14). Principal coordinate analysis (PCoA) of unweighted Unifrac phylogenetic distances also showed no significant changes in microbiota communities following 14 days milk-feeding (Fig. 1B).

### Comparison of overall community structure with antibiotic exposure

**Figure 3:**
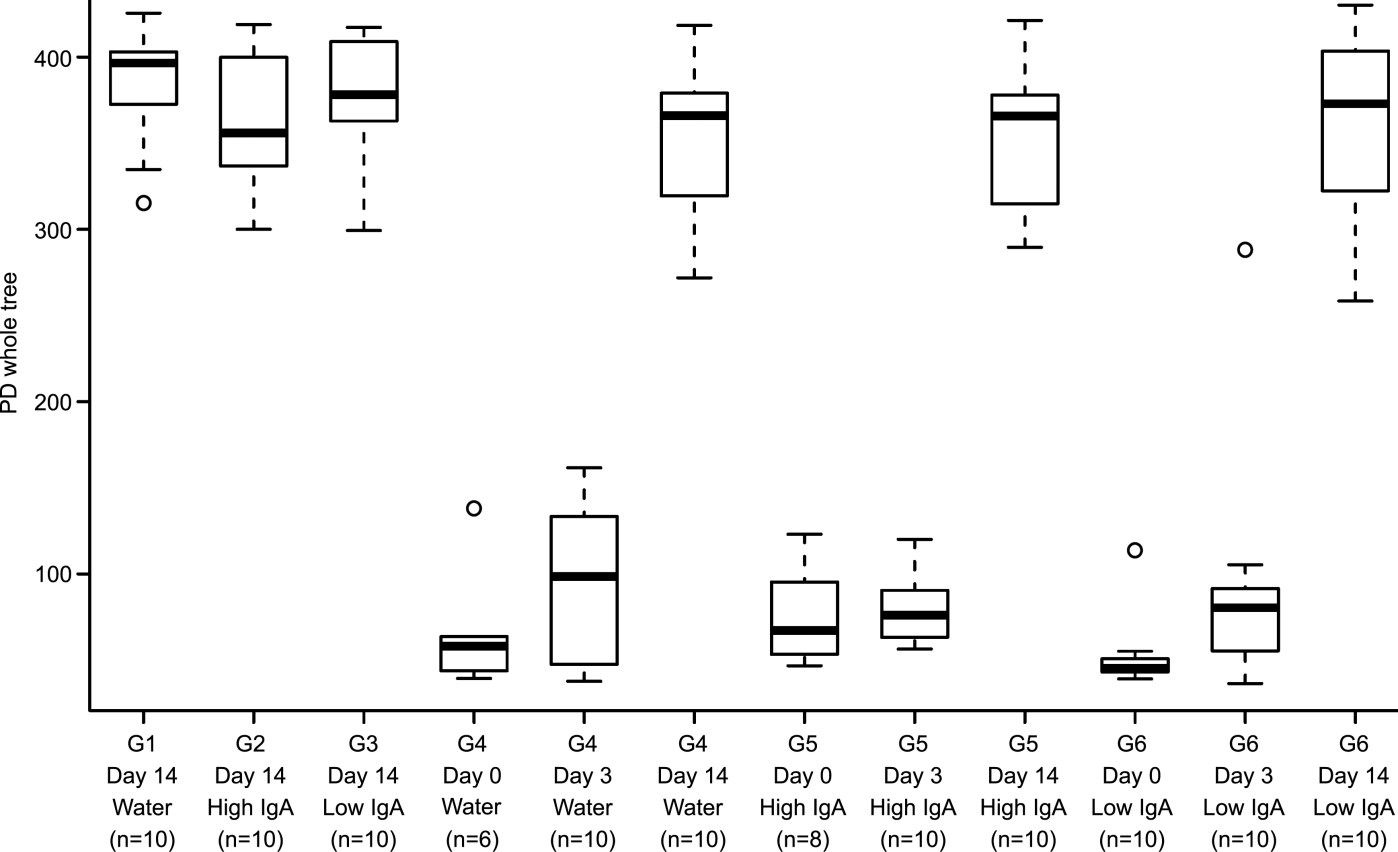
Diversity analysis of bacterial communities in response to treatments. The diversity of the bacterial community in faecal pellets was assessed for individual mice within each treatment group; Group 1 (no antibiotics, water), Group 2 (no antibiotics, High-IgA milk), Group 3 (no antibiotics, Low-IgA milk), Group 4 (antibiotics, water), Group 5 (antibiotics, High-IgA milk) and Group 6 (antibiotics, Low-IgA milk). For Groups 1 –3, data are shown for samples collected prior to water/milk feeding (Pre), and for samples collected at day 14 of the water/milk treatment period. For Groups 4–6, data are shown for samples collected at the beginning of the experiment (Pre), immediately after five days of antibiotic exposure at day 0, then at day 3 and day 14 of the water/milk treatment period. Boxes represent medians (25th–75th percentiles), whiskers represent 5th–95th percentiles, and open circles indicating outliers.

Groups exposed to ampicillin for five days showed a marked change in their microbiota, leading to communities consisting mainly of *Tenericutes*, *Firmicutes*, and *Proteobacteria*, with mean relative abundances of 76%, 12%, and 9%, respectively (Fig. 2A, Day 0). These compositional changes were also associated with a precipitous drop in community diversity (Fig. 3), consistent with expectations for the effects of an antibiotic.

Following antibiotic exposure and with 3 days of the water/milk treatments, there was a further change in community compositions, with a large decline in *Tenericutes* and concomitant expansion in *Firmicutes* and *Proteobacteria* (Fig. 2A, day 3). Although groups’ communities were still highly variable, some differences were observed between treatment groups at day 3; *Proteobacteria* proportions were significantly lower (*P* < 0.001) in mice given water (percent ± SEM: Water, 2.02 ± 1.42; High-IgA milk, 31.2 ± 7.9; Low-IgA milk, 46.1 ± 9.8), whereas *Bacteroidetes* were significantly lower in the Low-IgA milk group (*P* = 0.03) compared to the Water and High-IgA milk groups (Low-IgA milk, 8.2 ± 8.1; Water, 25.9 ± 10.5; High-IgA milk, 13.5 ± 7.0). PCoA of unweighted Unifrac phylogenetic distances also showed that antibiotic exposure still had a substantial effect on microbial community composition on day 3 (Fig. 2B). Similar to day 0, community diversity was still low at day 3 compared to pre-sample (Fig. 3).

**Figure 4:**
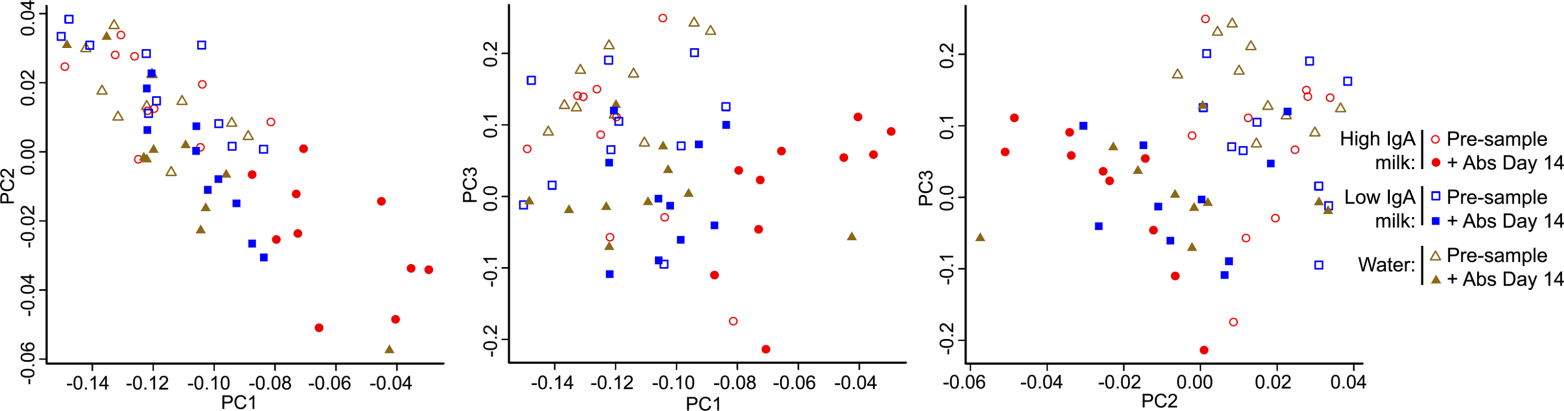
Comparison of bacterial communities before and after antibiotic exposure. Principle Co-ordinate plots (PC1 versus PC2, PC1 versus PC3 and PC2 versus PC3) for 16S rDNA sequencing data of bacterial communities in faecal pellets collected from individual mice that were exposed to antibiotics and then fed water/milk for a period of 14 days are shown for Group 4 (water, brown triangles), Group 5 (High-IgA milk, red circles) and Group 6 (Low-IgA milk, blue squares). Data are for samples collected at the beginning of the experiment prior to antibiotic exposure (Pre, open symbols) and then at Day 14 of the water/milk treatments (solid symbols).

By day 14 of the water/milk treatments, the bacterial populations were once again dominated by *Bacteriodetes* and *Firmicutes*, similar to the pre-samples collected before antibiotic exposure, and all antibiotic treated groups had a similar relative abundance of these phyla (Fig. 2A, Day 14). PCoA of unweighted Unifrac phylogenetic distances at day 14 showed that microbial communities in those groups exposed to antibiotics were similar to those groups not exposed to antibiotics (Fig. 2B). However, closer examination revealed that for the High-IgA milk group at day 14, microbial communities were more divergent from their pre sample compared with the Low-IgA milk or water groups at this time-point (Fig. 4). There were no differences observed in community diversity between antibiotic exposed groups at day 14 (*P* = 0.41, Fig. 3); diversity levels had recovered similar to pre-samples before antibiotic exposure and similar to levels of diversity for groups that had not received antibiotics.

### Progression of microbiota over time

A more detailed analysis at the lower taxonomic genus level was undertaken for individual mice within groups, using paired Wilcoxon Rank Sum tests, to follow change of microbiota over the 14 day feeding period. *P* values were adjusted for multiple testing using the Benjamini Hochberg false discovery rate (FDR) method. Taxa with an FDR <0.05 were considered significantly different.

#### No antibiotic exposure with 14 days milk feeding

In groups that had not received antibiotics, comparison of bacteria communities in pre-samples and day 14 samples showed there were no taxa at the genus level that differed in mean relative abundance with an FDR <0.05 (Table 1).

**Table 1:** Changes in taxa over time for groups not exposed to antibiotics and fed water/milks for a period of 14 days. Using paired Wilcoxon rank sum test, the relative mean level of each bacteria was compared between the pre-sample and Day 14 sample for individual mice within groups; Group 1 (water), Group 2 (High-IgA milk) and Group 3 (Low-IgA milk). Taxa with an FDR <0.05 were considered significantly different.

Group	Phylum	Genus or lowest identified taxonomic level	Pre mean	Pre sem	Day-14 mean	Day-14 sem	*P* value	*P* value FDR	Differ^*^
Group 1 (Water)	*Firmicutes*	Unclassified *Lactobacillaceae*	**0.50**	0.10	**1.73**	0.41	0.002	0.081	−1.23
	*Firmicutes*	*Lactobacillus*	**3.31**	0.57	**10.23**	2.23	0.004	0.081	−6.92
	*Firmicutes*	Unclassified *Lactobacillales*	**0.40**	0.07	**1.76**	0.35	0.004	0.081	−1.36
	*Firmicutes*	Unclassified *Bacilli*	**0.01**	0.00	**0.06**	0.01	0.010	0.126	−0.04
	*Firmicutes*	*Robinsoniella*	**0.04**	0.03	**0.21**	0.11	0.014	0.126	−0.17
	Unclassified	Unclassified Bacteria	**1.86**	0.23	**3.01**	0.24	0.014	0.126	−1.15
	*Proteobacteria*	*Parasutterella*	**0.02**	0.01	**0.01**	0.00	0.014	0.126	0.01
	*Bacteroidetes*	Unclassified *Bacteroidales*	**7.24**	0.71	**5.19**	0.56	0.027	0.212	2.05
	*Firmicutes*	Unclassified *Clostridiales*	**4.74**	0.92	**7.50**	1.38	0.037	0.256	−2.76
	*Firmicutes*	*Acetivibrio*	**0.01**	0.00	**0.03**	0.01	0.044	0.266	−0.02
Group 2 (High-IgA milk)	*Firmicutes*	*Papillibacter*	**0.14**	0.02	**0.05**	0.01	0.002	0.060	0.09
	*Firmicutes*	Unclassified *Clostridia*	**0.07**	0.01	**0.03**	0.01	0.002	0.060	0.04
	*Bacteroidetes*	*Barnesiella*	**5.87**	0.61	**8.56**	0.68	0.010	0.199	−2.69
	*Bacteroidetes*	Unclassified *Porphyromonadaceae*	**26.39**	2.22	**22.07**	1.42	0.020	0.298	4.32
	Firmicutes	Unclassified *Ruminococcaceae*	**3.38**	0.33	**2.01**	0.36	0.027	0.334	1.37
	*Proteobacteria*	*Desulfovibrio*	**0.11**	0.04	**0.03**	0.01	0.037	0.377	0.08
	*Firmicutes*	*Oscillibacter*	**0.93**	0.13	**0.50**	0.15	0.049	0.426	0.43
Group 3 (Low-IgA milk)	Unclassified	Unclassified Bacteria	**2.13**	0.21	**3.00**	0.15	0.006	0.298	0.09
	*Bacteroidetes*	*Prevotella*	**1.05**	0.29	**3.14**	0.43	0.010	0.298	−0.87
	*Bacteroidetes*	Unclassified *Prevotellaceae*	**1.77**	0.81	**4.20**	0.80	0.020	0.366	−2.08
	*Firmicutes*	*Ruminococcus*	**0.03**	0.01	**0.20**	0.06	0.027	0.366	−2.42
	*Firmicutes*	*Sporobacter*	**0.01**	0.00	**0.03**	0.01	0.030	0.366	−0.17
	*Bacteroidetes*	Unclassified *Porphyromonadaceae*	**28.17**	3.30	**21.30**	1.78	0.049	0.491	−0.02

**Notes.**

* Difference in the mean value for Pre and Day 14 samples.

#### Day 3 of water/milk treatments after antibiotic exposure

In groups that were exposed to antibiotics, marked differences in microbiota at the genus level at day 3 following antibiotic exposure compared to their state prior to antibiotics were observed, as previously noted at the phylum level. In mice fed water, the largest difference observed at day 3 was a relative increase in *Mycoplasma* proportions compared to pre-samples, with an increase from 0.08% to 20.1% (FDR = 0.033, Table 2). There was also an increase in *Paenibacillus* from 0.00 to 8.08% (FDR = 0.006) over the same time period. Concomitant with this was a decrease in Unclassified *Porphyromonadaceae* (32.25%–1.36%, FDR = 0.006), *Akkermansia* (7.69%–0.05%, FDR = 0.012), Unclassified *Lachnospiraceae* (7.67%–0.76%, FDR = 0.006), Unclassified *Bacteroidales* (7.68%–0.56%, FDR = 0.006) and *Barnesiella* (7.35%–0.20%, FDR = 0.006).

**Table 2:** Changes in taxa over time for groups exposed to antibiotics and fed water/milks for a period of 14 days. Using paired Wilcoxon rank sum test, the relative mean level of each bacteria was compared between the pre-sample and Day 3 sample for individual mice within groups; Group 4 (water), Group 5 (High-IgA milk) and Group 6 (Low-IgA milk). Taxa with differences greater than 1% are listed. Taxa with an FDR <0.05 were considered significantly different.

Group	Phylum	Genus or lowest identified taxonomic level	**Pre mean**	Pre sem	**Day-3 mean**	Day-3 sem	*P* value	*P* value FDR	Differ^*^
Group 4 (Water)	*Bacteroidetes*	Unclassified *Bacteroidales*	**7.68**	0.82	**0.56**	0.27	0.002	0.006	−7.12
	*Bacteroidetes*	*Barnesiella*	**7.35**	0.41	**0.20**	0.12	0.002	0.006	−7.15
	*Bacteroidetes*	Unclassified *Porphyromonadaceae*	**33.25**	1.84	**1.36**	0.81	0.002	0.006	−31.88
	*Bacteroidetes*	Unclassified *Prevotellaceae*	**3.10**	0.80	**0.00**	0.00	0.002	0.006	−3.09
	*Bacteroidetes*	*Prevotella*	**2.41**	0.26	**0.00**	0.00	0.002	0.006	−2.41
	*Bacteroidetes*	Unclassified *Bacteroidetes*	**1.90**	0.28	**0.04**	0.02	0.002	0.006	−1.86
	*Firmicutes*	*Paenibacillus*	**0.00**	0.00	**8.07**	7.47	0.002	0.006	8.07
	*Firmicutes*	Unclassified *Lachnospiraceae*	**7.67**	1.79	**0.76**	0.31	0.002	0.006	−6.91
	*Firmicutes*	Unclassified *Ruminococcaceae*	**2.19**	0.42	**0.03**	0.01	0.002	0.006	−2.16
	*Verrucomicrobia*	*Akkermansia*	**7.69**	1.74	**0.05**	0.04	0.004	0.012	−7.64
	*Firmicutes*	*Clostridium*	**0.00**	0.00	**3.45**	3.24	0.009	0.021	3.45
	*Proteobacteria*	*Escherichia/Shigella*	**0.00**	0.00	**1.42**	1.02	0.014	0.025	1.42
	*Tenericutes*	*Mycoplasma*	**0.08**	0.04	**20.10**	11.99	0.020	0.033	20.02
	*Firmicutes*	*Enterococcus*	**0.05**	0.01	**1.13**	0.58	0.027	0.039	1.08
Group 5 (High-IgA Milk)	*Bacteroidetes*	Unclassified *Bacteroidales*	**5.85**	0.68	**0.17**	0.08	0.002	0.004	−5.68
	*Bacteroidetes*	*Barnesiella*	**8.08**	1.33	**0.00**	0.00	0.002	0.004	−8.07
	*Bacteroidetes*	*Odoribacter*	**1.04**	0.20	**0.00**	0.00	0.002	0.004	−1.04
	*Bacteroidetes*	Unclassified *Porphyromonadaceae*	**26.83**	2.86	**0.00**	0.00	0.002	0.004	−26.82
	*Bacteroidetes*	*Parabacteroides*	**1.13**	0.22	**0.00**	0.00	0.002	0.004	−1.13
	*Bacteroidetes*	Unclassified *Prevotellaceae*	**1.83**	0.45	**0.00**	0.00	0.002	0.004	−1.83
	*Bacteroidetes*	*Prevotella*	**2.11**	0.48	**0.00**	0.00	0.002	0.004	−2.11
	*Bacteroidetes*	*Alistipes*	**1.45**	0.23	**0.00**	0.00	0.002	0.004	−1.45
	*Bacteroidetes*	Unclassified *Bacteroidetes*	**1.80**	0.20	**0.02**	0.01	0.002	0.004	−1.78
	*Firmicutes*	*Enterococcus*	**0.06**	0.02	**15.91**	3.95	0.002	0.004	15.85
	*Firmicutes*	Unclassified *Enterococcaceae*	**0.02**	0.00	**2.19**	0.49	0.002	0.004	2.18
	*Firmicutes*	Unclassified *Lactobacillales*	**0.62**	0.14	**15.36**	2.57	0.002	0.004	14.74
	*Firmicutes*	*Dorea*	**1.20**	0.32	**0.00**	0.00	0.002	0.004	−1.20
	*Firmicutes*	Unclassified *Lachnospiraceae*	**14.04**	3.67	**0.05**	0.05	0.002	0.004	−13.99
	*Firmicutes*	Unclassified *Clostridiales*	**7.90**	1.55	**0.07**	0.06	0.002	0.004	−7.82
	*Firmicutes*	Unclassified *Ruminococcaceae*	**3.58**	0.37	**0.00**	0.00	0.002	0.004	−3.58
	*Proteobacteria*	*Escherichia*/*Shigella*	**0.00**	0.00	**4.66**	2.58	0.002	0.004	4.66
	*Proteobacteria*	Unclassified *Enterobacteriaceae*	**0.00**	0.00	**22.11**	6.83	0.002	0.004	22.11
	*Proteobacteria*	*Raoultella*	**0.00**	0.00	**2.68**	1.04	0.002	0.004	2.68
	*Tenericutes*	*Mycoplasma*	**0.03**	0.03	**2.41**	0.92	0.002	0.004	2.37
	*Verrucomicrobia*	*Akkermansia*	**3.06**	1.20	**0.00**	0.00	0.009	0.015	−3.06
Group 6 (Low-IgA milk)	*Bacteroidetes*	*Parabacteroides*	**1.17**	0.34	**0.08**	0.08	0.002	0.006	−1.09
	*Bacteroidetes*	*Prevotella*	**1.33**	0.29	**0.22**	0.22	0.002	0.006	−1.11
	*Firmicutes*	Unclassified *Lachnospiraceae*	**15.35**	3.37	**2.08**	1.15	0.002	0.006	−13.28
	*Firmicutes*	Unclassified *Clostridiales*	**8.12**	1.63	**2.54**	1.52	0.002	0.006	−5.57
	*Firmicutes*	Unclassified *Ruminococcaceae*	**3.11**	0.49	**0.03**	0.03	0.002	0.006	−3.07
	*Proteobacteria*	*Serratia*	**0.00**	0.00	**6.34**	4.06	0.002	0.006	6.34
	*Tenericutes*	*Mycoplasma*	**0.04**	0.03	**1.12**	0.32	0.002	0.006	1.09
	*Bacteroidetes*	Unclassified *Porphyromonadaceae*	**31.90**	3.32	**2.81**	2.81	0.004	0.010	−29.08
	*Bacteroidetes*	Unclassified *Bacterioidetes*	**1.84**	0.31	**0.23**	0.22	0.004	0.010	−1.61
	*Firmicutes*	*Enterococcus*	**0.05**	0.01	**9.18**	2.75	0.004	0.010	9.13
	*Proteobacteria*	*Escherichia/Shigella*	**0.00**	0.00	**9.09**	4.15	0.004	0.010	9.09
	*Firmicutes*	Unclassified *Enterococcaceae*	**0.00**	0.00	**1.50**	0.41	0.009	0.017	1.50
	*Proteobacteria*	Unclassified *Enterobacteriaceae*	**0.00**	0.00	**25.36**	8.09	0.009	0.017	25.36
	*Proteobacteria*	*Raoultella*	**0.00**	0.00	**3.81**	2.32	0.009	0.017	3.81
	*Bacteroidetes*	*Barnesiella*	**4.99**	0.59	**0.90**	0.89	0.010	0.017	−4.09
	*Bacteroidetes*	*Alistipes*	**1.32**	0.20	**0.26**	0.26	0.010	0.017	−1.06
	*Firmicutes*	Unclassified *Lactobacillales*	**0.51**	0.14	**11.83**	2.74	0.010	0.017	11.32
	*Bacteroidetes*	Unclassified *Bacteroidales*	**6.51**	0.68	**1.12**	1.12	0.020	0.031	−5.39

**Notes.**

* Difference in the mean value for Pre and Day 14 samples.

Similar to the water-fed group, mice fed Low-IgA milk also showed a large decrease in Unclassified *Porphyromonadaceae* at day 3 compared to pre-samples (31.9%–2.81%, FDR = 0.01, Table 2). The Low-IgA milk group also had a relative reduction in Unclassified *Lachnospiraceae* (15.35%–2.08%, FDR = 0.006), *Unclassified Clostridiales* (8.12 to 2.54, FDR = 0.006), *Unclassified Bacteroidales* (6.51 to 1.12, FDR = 0.30) and *Barnsiella* (4.99 to 0.90, FDR = 0.017). Bacteria which showed a substantial increase in abundance at day 3 compared to pre-samples in the Low-IgA milk group included Unclassified *Enterobacteriaceae* (0.0%–25.36%. FDR = 0.017), Unclassified *Lactobacillales* (0.51%–11.83%, FDR = 0.017), *Enterococcus* (0.05%–9.18%, FDR = 0.010), *Escherichia*/*Shigella* (0.0%–9.09%.FDR = 0.010) and *Serratia* (0.0 to 6.34, FDR = 0.006).

Comparing day 3 to pre-sample, mice fed High-IgA milk showed a similar progression to mice fed Low-IgA milk (Table 2); Unclassified *Porphyromonadaceae* (26.83%–0.0%, FDR = 0.004), Unclassified *Lachnospiraceae* (14.04%–0.05%, FDR = 0.004), Unclassified *Clostridiales* (7.90 to 0.07, FDR = 0.004), *Unclassified Bacteroidales* (5.85 to 0.17, FDR = 0.0.004) and *Barnsiella* (8.08 to 0.00, FDR = 0.004) all decreased. Similarly Unclassified *Enterobacteriaceae* (0.0%–22.11%, FDR = 0.017), Unclassified *Lactobacillales* (0.62%–15.36%, FDR = 0.004) and *Enterococcus* (0.06%–15.91%, FDR = 0.004) were increased.

#### Day 14 of water/milk treatments after antibiotic exposure

By day 14, mice that were fed water had faecal communities that were very similar to the pre-sample state, with no taxa that differed with an FDR <0.05 (Table 3). However, at this time point, mice that were fed High-IgA or Low-IgA milks showed some taxa that were significantly different to pre-sample taxa (FDR < 0.05; Table 3), in contrast to mice that were not exposed to antibiotics and fed milks (Table 1).

**Table 3:** Changes in taxa over time for groups exposed to antibiotics and fed water/milks for a period of 14 days. Using paired Wilcoxon rank sum test, the relative mean level of each bacteria was compared between the pre-sample and Day 14 sample for individual mice within groups; Group 4 (water), Group 5 (High-IgA milk) and Group 6 (Low-IgA milk). Taxa with an FDR <0.05 were considered significantly different.

Group	Phylum	Genus or lowest identified taxonomic level	**Pre mean**	Pre sem	**Day-14 mean**	Day-14 sem	*P* value	*P* value FDR	Differ^*^
Group 4 (Water)	*Bacteroidetes*	Unclassified *Prevotellaceae*	**3.10**	0.80	**0.98**	0.17	0.002	0.075	−2.12
	*Bacteroidetes*	*Prevotella*	**2.41**	0.26	**1.19**	0.26	0.004	0.075	−1.23
	*Firmicutes*	*Streptococcus*	**0.02**	0.00	**0.00**	0.00	0.009	0.075	−0.02
	*Proteobacteria*	*Helicobacter*	**0.28**	0.13	**0.00**	0.00	0.009	0.075	−0.28
	*Firmicutes*	Unclassified *Lactobacillaceae*	**0.38**	0.08	**1.60**	0.48	0.010	0.075	1.21
Group 5 (High-IgA milk)	*Firmicutes*	Unclassified *Bacilli*	**0.01**	0.00	**0.07**	0.01	0.002	0.020	0.07
	Unclassified Bacteria	Unclassified Bacteria	**2.01**	0.21	**3.29**	0.17	0.002	0.020	1.29
	*Proteobacteria*	*Desulfovibrio*	**0.08**	0.02	**0.00**	0.00	0.002	0.020	−0.08
	*Proteobacteria*	Unclassified *Desulfovibrionales*	**0.22**	0.07	**0.02**	0.01	0.002	0.020	−0.20
	*Proteobacteria*	*Helicobacter*	**0.43**	0.20	**0.00**	0.00	0.002	0.020	−0.43
	*Proteobacteria*	Unclassified *Proteobacteria*	**0.09**	0.02	**0.02**	0.01	0.002	0.020	−0.08
	*Firmicutes*	Unclassified *Lactobacillales*	**0.62**	0.14	**2.03**	0.32	0.004	0.032	1.41
	*Bacteroidetes*	*Barnesiella*	**8.08**	1.33	**14.05**	1.80	0.006	0.032	5.98
	*Bacteroidetes*	*Rikenella*	**0.51**	0.17	**0.06**	0.03	0.006	0.032	−0.45
	*Firmicutes*	*Lactobacillus*	**4.50**	0.79	**12.06**	1.83	0.006	0.032	7.57
	*Firmicutes*	Unclassified *Firmicutes*	**0.61**	0.10	**1.43**	0.14	0.006	0.032	0.81
	*Deferribacteres*	*Mucispirillum*	**0.16**	0.07	**0.00**	0.00	0.009	0.045	−0.16
	*Firmicutes*	Unclassified *Lactobacillaceae*	**0.52**	0.10	**1.69**	0.41	0.010	0.045	1.16
	*Bacteroidetes*	Unclassified *Porphyromonadaceae*	**26.83**	2.86	**17.87**	0.58	0.014	0.059	−8.96
	*Bacteroidetes*	*Parabacteroides*	**1.13**	0.22	**0.37**	0.18	0.020	0.078	−0.76
	*Firmicutes*	*Gemella*	**0.03**	0.01	**0.00**	0.00	0.022	0.079	−0.03
	*Proteobacteria*	*Parasutterella*	**0.02**	0.01	**0.00**	0.00	0.022	0.079	−0.02
	*Proteobacteria*	Unclassified *Desulfovibrionaceae*	**0.05**	0.01	**0.02**	0.01	0.027	0.091	−0.04
	*Proteobacteria*	Unclassified *Helicobacteraceae*	**0.03**	0.01	**0.00**	0.00	0.036	0.101	−0.03
	*Tenericutes*	*Anaeroplasma*	**0.01**	0.01	**0.00**	0.00	0.036	0.101	−0.01
	*Firmicutes*	Unclassified *Ruminococcaceae*	**3.58**	0.37	**2.48**	0.34	0.037	0.101	−1.10
	*Firmicutes*	*Papillibacter*	**0.11**	0.01	**0.06**	0.01	0.037	0.101	−0.05
	*Bacteroidetes*	Unclassified *Bacteroidales*	**5.85**	0.68	**4.28**	0.35	0.049	0.127	−1.57
	*Actinobacteria*	Unclassified *Coriobacteriaceae*	**0.11**	0.02	**0.18**	0.04	0.064	0.161	0.07
	*Bacteroidetes*	*Bacteroides*	**6.95**	0.90	**4.89**	0.92	0.084	0.202	−2.06
	*Firmicutes*	*Oscillibacter*	**0.80**	0.22	**0.41**	0.12	0.105	0.243	−0.39
	*Actinobacteria*	*Enterorhabdus*	**0.09**	0.02	**0.15**	0.03	0.131	0.291	0.06
	*Actinobacteria*	*Olsenella*	**0.00**	0.00	**0.02**	0.01	0.178	0.374	0.01
	*Firmicutes*	*Coprococcus*	**0.02**	0.01	**0.03**	0.01	0.193	0.374	0.01
	*Firmicutes*	*Dorea*	**1.20**	0.32	**0.60**	0.13	0.193	0.374	−0.60
	*Firmicutes*	*Robinsoniella*	**0.50**	0.38	**0.06**	0.04	0.193	0.374	−0.44
	*Proteobacteria*	Unclassified *Alphaproteobacteria*	**0.03**	0.02	**0.01**	0.00	0.205	0.384	−0.02
	*Bacteroidetes*	*Odoribacter*	**1.04**	0.20	**0.69**	0.12	0.232	0.423	−0.35
	*Firmicutes*	Unclassified *Clostridiales*	**7.90**	1.55	**9.26**	1.38	0.275	0.472	1.37
	*Firmicutes*	*Acetivibrio*	**0.03**	0.01	**0.04**	0.01	0.275	0.472	0.01
	*Firmicutes*	*Johnsonella*	**0.04**	0.01	**0.03**	0.01	0.294	0.478	−0.02
	*Firmicutes*	*Parasporobacterium*	**0.01**	0.00	**0.00**	0.00	0.295	0.478	0.00
	*Bacteroidetes*	*Prevotella*	**2.11**	0.48	**1.34**	0.44	0.322	0.496	−0.76
	*Bacteroidetes*	*Alistipes*	**1.45**	0.23	**2.09**	0.36	0.322	0.496	0.64
Group 6 (Low-IgA milk)	*Bacteroidetes*	*Barnesiella*	**4.99**	0.59	**12.41**	1.78	0.002	0.027	7.42
	*Bacteroidetes*	*Rikenella*	**0.40**	0.12	**0.01**	0.00	0.002	0.027	−0.39
	*Proteobacteria*	*Desulfovibrio*	**0.12**	0.03	**0.00**	0.00	0.002	0.027	−0.12
	*Proteobacteria*	Unclassified *Desulfovibrionales*	**0.19**	0.03	**0.03**	0.01	0.002	0.027	−0.15

**Notes.**

* Difference in the mean value for Pre and Day 14 samples.

In mice fed Low-IgA milk after antibiotic exposure, four taxa remained significantly different (FDR <0.05) at day 14 compared to their pre-sample communities (Table 3). Of these taxa, *Barnesiella* showed the largest changes which increase from 4.99% of the community in the pre-samples to 12.41% at day 14.

Mice that were fed High-IgA milk after antibiotic exposure showed 13 taxa that were significantly different between their pre-sample faecal communities and day 14 samples (Table 3). Among these taxa, *Lactobacillus* increased from 4.5% to 12.06%. Similarly to the Low-IgA milk group, mice fed High-IgA milk also showed a significant increase in *Barnesiella*, increasing from 8.08% in their pre-sample community to 14.05% at day 14.

## Discussion

The microbiota profile we observed in mice before treatments was similar to that described in the mammalian gastrointestinal tract by others (Eckburg et al., 2005), with the phyla Firmicutes and Bacteroides dominating, and Proteobacteria, and Actinobacteria less abundant. Antibiotic exposure resulted in considerable alteration to the profile of the microbiota and its diversity, as reported by others (Hill et al., 2010; Ubeda et al., 2010), and the microbiota was still in a state of flux three days after antibiotic withdrawal, with high variability between individuals and also variability between groups with different treatments of water and milks. By day 14, the microbiota was similar to the pre-antibiotic state, although some differences were observed between groups.

With no antibiotic exposure, High-IgA or Low-IgA milks had no discernible effects on the intestinal microbiota in mice. However, after perturbation of the microbiota with antibiotics, feeding milk did alter how the microbial communities recovered. In contrast, mice that were exposed to antibiotics and then fed water had microbiota compositions at day 14 that were similar to their microbiota composition prior to antibiotics. A recent publication showed that antibiotics do not perturb the gut IgA compartment and that there is a longitudinal persistence of memory B cells (Lindner et al., 2015). This suggests that following antibiotics, host-derived IgA remains unchanged and drives re-colonisation of microbiota to the pre-antibiotic state, as we observed with the water-fed group. Yet, adding milk to the diet changed the environment for re-colonisation of bacteria. To our knowledge, this study is the first to report that ingestion of cows’ milk affects the balance of microbiota present in the mouse intestine following antibiotic exposure.

The divergent effects of feeding milk on microbiota following antibiotic exposure was evident at day 3 of feeding, and the differences persisted to day 14. Mice that were given water showed a significant increase in *Mycoplasma* at day 3 that was not observed in the milk-fed mice. While mycoplasma infection is more commonly associated with respiratory disease (Taylor-Robinson & Bebear, 1997), *Mycoplasma* has also been implicated in Crohn’s disease (Roediger & Macfarlane, 2002). *Mycoplasma* lack rigid cell walls and are, therefore, resistant to antibiotics that act on these structures, such as ampicillin, the antibiotic used in this study (Hayes et al., 1993; Martens et al., 1990). When microbial groups are removed from a system there is the potential for other groups to fill in the gaps; success of these in-fillers may be dependent on available substrates. By adding milk to the system, we potentially provided additional substrates for bacteria. Equally, because the milks were not pasteurised, they may have provided a source of milk-derived bacteria that competed with the *Mycoplasma*. Further study would be required to elucidate the mechanism behind the suppression of *Mycoplasma* at day 3 in the milk-fed mice.

Ampicillin is a broad spectrum antibiotic with activity against gram-positive bacteria and some groups of gram-negative bacteria including some *Proteobacteria*. In the milk-fed groups that were exposed to antibiotics, the relative proportion of *Proteobacteria* were higher on day 3 compared with the water-fed groups. Higher levels of these bacteria are not desirable and are increasingly being recognized as signatures of an unstable (dysbiotic) community (Shin, Whon & Bae, 2015). However this was a transient effect, and by day 14 of milk-feeding the relative levels of *Proteobacteria* were reduced and similar to their pre-antibiotic levels.

Our study showed other potentially beneficial effects of feeding milk after antibiotic use. *Barnesiella* were significantly elevated in mice fed the High-IgA and Low-IgA milks for 14 days after antibiotic exposure, compared to mice from the water-fed group. Characterization of the faecal microbiota of patients undergoing transplantation demonstrated that intestinal colonization with *Barnesiella* conferred resistance to pathogenic infection. The studies indicated that bacteria belonging to the *Barnesiella* genus may provide novel approaches to prevent the spread of highly antibiotic-resistant bacteria (Ubeda et al., 2010). We also showed that feeding High-IgA milk was correlated with increased *Lactobacillus* at day 14. Lactobacilli have been associated with numerous beneficial properties such as reducing intestinal inflammation (Bruzzese et al., 2014) and improving resistance to infection by *Clostridium difficile*, a pathogen with significant negative health impacts in the human population (Schubert, Sinani & Schloss, 2015). The cause of the differential effects between Low-IgA milk and High-IgA milk may have been due to the levels of IgA; on the other hand, there may be have been other components present in milks from cows that produce high IgA levels that contributed to or provided the beneficial effect. These components may include IgG, lactoferrin and oligosaccharides.

The variation in effects we observed between milk-fed groups and water-fed groups are not sufficient by themselves to make definitive conclusions on the health benefits of ingesting milk, especially as the mouse model is not always translatable to the situation in the human gut. We also observed marked variability, both within the treatment groups and between the effects over time and measuring the microbiota at a further time point of 30 or 60 days would have provided more information about whether the effects were stable or transient. Another limitation is that these studies were performed with raw, unprocessed milk; milk-borne bacteria may have contributed to our findings that the microbiota of milk-fed groups recovered differently from antibiotic exposure compared to the group fed water. Differences in bacteria in High-IgA and Low-IgA milks may also have contributed to the differential effects observed with these milks, although, the bacteria found in milk should be similar from healthy animals housed on the same farm. Milk components in dairy foods also undergo some modification during processing and manufacturing procedures. Immunoglobulins are among the more thermolabile milk proteins and exposure to processing operations such as heat, pressure, or pH change can affect the conformation of these proteins and ultimately their antibody activity (reviewed in Hurley & Theil (2011)). However, using lower temperatures and longer retention times is an effective way of improving the quality of heat-treated milk (Czank et al., 2009). The mechanisms giving rise to our observed effects of milk, and the specific molecules in milk that are responsible, will be the subject of future studies.

## Conclusion

Exposure of mice to ampicillin for five days results in profound changes to their intestinal microbiota, involving a transient loss of bacterial diversity, as expected. The recovery to a state resembling that prior to exposure to antibiotic occurred between day 3 and day 14 after antibiotic use. Feeding mice cows’ milk as their sole source of liquid during the recovery period, was associated with an altered balance of microbial communities in the gut compared with feeding water. Feeding milk containing high levels of IgA correlated with some differences in the prevalence of individual bacterial groups, compared with milk containing low levels of IgA. Overall, these findings add to a knowledge platform for optimising intestinal function after treatment with antibiotics in the human population.

##  Supplemental Information

10.7717/peerj.2518/supp-1 Table S1 Supplementary data: Changes in taxa over time for groups exposed to antibiotics and fed water/milks for a period of 14 days Using paired Wilcoxon rank sum test, the relative mean level of each bacteria was compared between the pre-sample and Day 3 sample for individual mice within groups; Group 4 (water), Group 5 (High-IgA milk) and Group 6 (Low-IgA milk). All taxa are listed. Taxa with an FDR <0.05 were considered significantly different.

## Additional Information and Declarations

### Data Deposition

The following information was supplied regarding data availability: NCBI SRA accession number SRP076637.
