# Supplementary material for: Feeding bovine milks with low or high IgA levels is associated with altered re-establishment of murine intestinal microbiota after antibiotic treatment
Source: PeerJ. 2016 Sep 29;4:e2518. doi: 10.7717/peerj.2518 (PMC5047218; doi:10.7717/peerj.2518)
Supplement: Table S1 — Using paired Wilcoxon rank sum test, the relative mean level of each bacteria was compared between the pre-sample and Day 3 sample for individual mice within groups; Group 4 (water), Group 5 (High-IgA milk) and Group 6 (Low-IgA milk). All taxa are listed. Taxa with an FDR <0.05 were considered significantly different. [file peerj-04-2518-s001.docx]

| Group | Phylum | Genus or lowest identified taxonomic level | **Pre mean** | Pre sem | **Day-3 mean** | Day-3 sem | P value | P value FDR | Differ* |
| --- | --- | --- | --- | --- | --- | --- | --- | --- | --- |
| Group 4 (Water) | *Actinobacteria* | Unclassified *Coriobacteriaceae* | **0.11** | 0.02 | **0.00** | 0.00 | 0.002 | 0.006 | -0.10 |
|  | *Bacteroidetes* | Unclassified *Bacteroidales* | **7.68** | 0.82 | **0.56** | 0.27 | 0.002 | 0.006 | -7.12 |
|  | *Bacteroidetes* | *Barnesiella* | **7.35** | 0.41 | **0.20** | 0.12 | 0.002 | 0.006 | -7.15 |
|  | *Bacteroidetes* | *Odoribacter* | **0.73** | 0.20 | **0.00** | 0.00 | 0.002 | 0.006 | -0.72 |
|  | *Bacteroidetes* | Unclassified *Porphyromonadaceae* | **33.25** | 1.84 | **1.36** | 0.81 | 0.002 | 0.006 | -31.88 |
|  | *Bacteroidetes* | *Parabacteroides* | **1.20** | 0.22 | **0.00** | 0.00 | 0.002 | 0.006 | -1.20 |
|  | *Bacteroidetes* | Unclassified *Prevotellaceae* | **3.10** | 0.80 | **0.00** | 0.00 | 0.002 | 0.006 | -3.09 |
|  | *Bacteroidetes* | *Prevotella* | **2.41** | 0.26 | **0.00** | 0.00 | 0.002 | 0.006 | -2.41 |
|  | *Bacteroidetes* | *Rikenella* | **0.23** | 0.05 | **0.00** | 0.00 | 0.002 | 0.006 | -0.23 |
|  | *Bacteroidetes* | Unclassified *Bacteroidetes* | **1.90** | 0.28 | **0.04** | 0.02 | 0.002 | 0.006 | -1.86 |
|  | *Firmicutes* | *Paenibacillus* | **0.00** | 0.00 | **8.07** | 7.47 | 0.002 | 0.006 | 8.07 |
|  | *Firmicutes* | Unclassified *Enterococcaceae* | **0.01** | 0.00 | **0.32** | 0.14 | 0.002 | 0.006 | 0.31 |
|  | *Firmicutes* | Unclassified *Lachnospiraceae* | **7.67** | 1.79 | **0.76** | 0.31 | 0.002 | 0.006 | -6.91 |
|  | *Firmicutes* | Oscillibacter | **0.35** | 0.09 | **0.00** | 0.00 | 0.002 | 0.006 | -0.34 |
|  | *Firmicutes* | Unclassified *Ruminococcaceae* | **2.19** | 0.42 | **0.03** | 0.01 | 0.002 | 0.006 | -2.16 |
|  | *Firmicutes* | *Papillibacter* | **0.08** | 0.01 | **0.00** | 0.00 | 0.002 | 0.006 | -0.08 |
|  | *Firmicutes* | *Sporobacter* | **0.04** | 0.01 | **0.00** | 0.00 | 0.002 | 0.006 | -0.04 |
|  | *Proteobacteria* | *Desulfovibrio* | **0.08** | 0.03 | **0.00** | 0.00 | 0.002 | 0.006 | -0.08 |
|  | *Proteobacteria* | Unclassified *Desulfovibrionales* | **0.15** | 0.04 | **0.00** | 0.00 | 0.002 | 0.006 | -0.15 |
|  | *Proteobacteria* | Unclassified *Proteobacteria* | **0.15** | 0.03 | **0.00** | 0.00 | 0.002 | 0.006 | -0.14 |
|  | *Firmicutes* | Unclassified *Bacilli* | **0.01** | 0.00 | **0.44** | 0.23 | 0.004 | 0.012 | 0.44 |
|  | *Verrucomicrobia* | *Akkermansia* | **7.69** | 1.74 | **0.05** | 0.04 | 0.004 | 0.012 | -7.64 |
|  | *Firmicutes* | *Streptococcus* | **0.02** | 0.00 | **0.00** | 0.00 | 0.009 | 0.021 | -0.02 |
|  | *Firmicutes* | *Clostridium* | **0.00** | 0.00 | **3.45** | 3.24 | 0.009 | 0.021 | 3.45 |
|  | *Firmicutes* | Unclassified *Clostridiaceae* | **0.00** | 0.00 | **0.85** | 0.81 | 0.009 | 0.021 | 0.85 |
|  | *Firmicutes* | *Dorea* | **0.30** | 0.11 | **0.00** | 0.00 | 0.009 | 0.021 | -0.30 |
|  | *Firmicutes* | *Butyricicoccus* | **0.05** | 0.02 | **0.00** | 0.00 | 0.009 | 0.021 | -0.05 |
|  | *Proteobacteria* | *Helicobacter* | **0.28** | 0.13 | **0.00** | 0.00 | 0.009 | 0.021 | -0.28 |
|  | *Firmicutes* | Unclassified *Bacillales* | **0.00** | 0.00 | **0.45** | 0.35 | 0.014 | 0.025 | 0.45 |
|  | *Firmicutes* | *Gemella* | **0.03** | 0.01 | **0.00** | 0.00 | 0.014 | 0.025 | -0.02 |
|  | *Firmicutes* | *Coprococcus* | **0.02** | 0.01 | **0.00** | 0.00 | 0.014 | 0.025 | -0.02 |
|  | *Firmicutes* | *Ruminococcus* | **0.44** | 0.12 | **0.00** | 0.00 | 0.014 | 0.025 | -0.44 |
|  | *Proteobacteria* | Unclassified *Alphaproteobacteria* | **0.03** | 0.01 | **0.00** | 0.00 | 0.014 | 0.025 | -0.03 |
|  | *Proteobacteria* | *Parasutterella* | **0.08** | 0.03 | **0.00** | 0.00 | 0.014 | 0.025 | -0.08 |
|  | *Proteobacteria* | Unclassified *Desulfovibrionaceae* | **0.04** | 0.02 | **0.00** | 0.00 | 0.014 | 0.025 | -0.04 |
|  | *Proteobacteria* | *Escherichia/Shigella* | **0.00** | 0.00 | **1.42** | 1.02 | 0.014 | 0.025 | 1.42 |
|  | *Proteobacteria* | Unclassified *Gammaproteobacteria* | **0.00** | 0.00 | **0.04** | 0.02 | 0.014 | 0.025 | 0.04 |
|  | *Tenericutes* | *Mycoplasma* | **0.08** | 0.04 | **20.10** | 11.99 | 0.020 | 0.033 | 20.02 |
|  | *Firmicutes* | *Marvinbryantia* | **0.02** | 0.01 | **0.00** | 0.00 | 0.022 | 0.034 | -0.02 |
|  | *Firmicutes* | Unclassified *Peptostreptococcaceae* | **0.00** | 0.00 | **0.95** | 0.48 | 0.022 | 0.034 | 0.95 |
|  | *Firmicutes* | *Acetivibrio* | **0.03** | 0.01 | **0.00** | 0.00 | 0.022 | 0.034 | -0.03 |
|  | *Proteobacteria* | Unclassified *Enterobacteriaceae* | **0.00** | 0.00 | **0.50** | 0.35 | 0.022 | 0.034 | 0.49 |
|  | *Tenericutes* | Unclassified *Mycoplasmataceae* | **0.00** | 0.00 | **0.41** | 0.25 | 0.022 | 0.034 | 0.41 |
|  | *Actinobacteria* | *Enterorhabdus* | **0.07** | 0.01 | **0.03** | 0.02 | 0.027 | 0.039 | -0.04 |
|  | *Firmicutes* | *Enterococcus* | **0.05** | 0.01 | **1.13** | 0.58 | 0.027 | 0.039 | 1.08 |
|  | *Firmicutes* | *Anaerosporobacter* | **0.00** | 0.00 | **0.77** | 0.57 | 0.036 | 0.050 | 0.77 |
|  | *Firmicutes* | *Johnsonella* | **0.02** | 0.01 | **0.00** | 0.00 | 0.036 | 0.050 | -0.02 |
| Group 5 (High-IgA Milk) | *Actinobacteria* | *Enterorhabdus* | **0.09** | 0.02 | **0.00** | 0.00 | 0.002 | 0.004 | -0.09 |
|  | *Actinobacteria* | Unclassified *Coriobacteriaceae* | **0.11** | 0.02 | **0.00** | 0.00 | 0.002 | 0.004 | -0.11 |
|  | *Bacteroidetes* | Unclassified *Bacteroidales* | **5.85** | 0.68 | **0.17** | 0.08 | 0.002 | 0.004 | -5.68 |
|  | *Bacteroidetes* | Barnesiella | **8.08** | 1.33 | **0.00** | 0.00 | 0.002 | 0.004 | -8.07 |
|  | *Bacteroidetes* | *Odoribacter* | **1.04** | 0.20 | **0.00** | 0.00 | 0.002 | 0.004 | -1.04 |
|  | *Bacteroidetes* | Unclassified *Porphyromonadaceae* | **26.83** | 2.86 | **0.00** | 0.00 | 0.002 | 0.004 | -26.82 |
|  | *Bacteroidetes* | *Parabacteroides* | **1.13** | 0.22 | **0.00** | 0.00 | 0.002 | 0.004 | -1.13 |
|  | *Bacteroidetes* | Unclassified *Prevotellaceae* | **1.83** | 0.45 | **0.00** | 0.00 | 0.002 | 0.004 | -1.83 |
|  | *Bacteroidetes* | *Prevotella* | **2.11** | 0.48 | **0.00** | 0.00 | 0.002 | 0.004 | -2.11 |
|  | *Bacteroidetes* | *Alistipes* | **1.45** | 0.23 | **0.00** | 0.00 | 0.002 | 0.004 | -1.45 |
|  | *Bacteroidetes* | *Rikenella* | **0.51** | 0.17 | **0.00** | 0.00 | 0.002 | 0.004 | -0.51 |
|  | *Bacteroidetes* | Unclassified *Bacteroidetes* | **1.80** | 0.20 | **0.02** | 0.01 | 0.002 | 0.004 | -1.78 |
|  | *Firmicutes* | Unclassified *Carnobacteriaceae* | **0.00** | 0.00 | **0.18** | 0.03 | 0.002 | 0.004 | 0.18 |
|  | *Firmicutes* | *Enterococcus* | **0.06** | 0.02 | **15.91** | 3.95 | 0.002 | 0.004 | 15.85 |
|  | *Firmicutes* | Unclassified *Enterococcaceae* | **0.02** | 0.00 | **2.19** | 0.49 | 0.002 | 0.004 | 2.18 |
|  | *Firmicutes* | Unclassified *Lactobacillales* | **0.62** | 0.14 | **15.36** | 2.57 | 0.002 | 0.004 | 14.74 |
|  | *Firmicutes* | Unclassified *Bacilli* | **0.01** | 0.00 | **0.17** | 0.04 | 0.002 | 0.004 | 0.16 |
|  | *Firmicutes* | *Dorea* | **1.20** | 0.32 | **0.00** | 0.00 | 0.002 | 0.004 | -1.20 |
|  | *Firmicutes* | Unclassified *Lachnospiraceae* | **14.04** | 3.67 | **0.05** | 0.05 | 0.002 | 0.004 | -13.99 |
|  | *Firmicutes* | Unclassified *Clostridiales* | **7.90** | 1.55 | **0.07** | 0.06 | 0.002 | 0.004 | -7.82 |
|  | *Firmicutes* | *Oscillibacter* | **0.80** | 0.22 | **0.00** | 0.00 | 0.002 | 0.004 | -0.79 |
|  | *Firmicutes* | Unclassified *Ruminococcaceae* | **3.58** | 0.37 | **0.00** | 0.00 | 0.002 | 0.004 | -3.58 |
|  | *Firmicutes* | *Papillibacter* | **0.11** | 0.01 | **0.00** | 0.00 | 0.002 | 0.004 | -0.11 |
|  | *Firmicutes* | *Ruminococcus* | **0.32** | 0.11 | **0.00** | 0.00 | 0.002 | 0.004 | -0.32 |
|  | *Firmicutes* | *Holdemania* | **0.08** | 0.02 | **0.00** | 0.00 | 0.002 | 0.004 | -0.08 |
|  | *Proteobacteria* | *Desulfovibrio* | **0.08** | 0.02 | **0.00** | 0.00 | 0.002 | 0.004 | -0.08 |
|  | *Proteobacteria* | Unclassified *Desulfovibrionaceae* | **0.05** | 0.01 | **0.00** | 0.00 | 0.002 | 0.004 | -0.05 |
|  | *Proteobacteria* | Unclassified *Desulfovibrionales* | **0.22** | 0.07 | **0.00** | 0.00 | 0.002 | 0.004 | -0.22 |
|  | *Proteobacteria* | *Helicobacter* | **0.43** | 0.20 | **0.00** | 0.00 | 0.002 | 0.004 | -0.43 |
|  | *Proteobacteria* | *Enterobacter* | **0.00** | 0.00 | **0.38** | 0.22 | 0.002 | 0.004 | 0.38 |
|  | *Proteobacteria* | *Escherichia/Shigella* | **0.00** | 0.00 | **4.66** | 2.58 | 0.002 | 0.004 | 4.66 |
|  | *Proteobacteria* | Unclassified *Enterobacteriaceae* | **0.00** | 0.00 | **22.11** | 6.83 | 0.002 | 0.004 | 22.11 |
|  | *Proteobacteria* | *Raoultella* | **0.00** | 0.00 | **2.68** | 1.04 | 0.002 | 0.004 | 2.68 |
|  | *Proteobacteria* | Unclassified *Gammaproteobacteria* | **0.00** | 0.00 | **0.08** | 0.01 | 0.002 | 0.004 | 0.08 |
|  | *Tenericutes* | *Mycoplasma* | **0.03** | 0.03 | **2.41** | 0.92 | 0.002 | 0.004 | 2.37 |
|  | *Firmicutes* | Unclassified *Clostridia* | **0.05** | 0.01 | **0.00** | 0.00 | 0.006 | 0.011 | -0.04 |
|  | *Firmicutes* | *Butyricicoccus* | **0.13** | 0.03 | **0.00** | 0.00 | 0.009 | 0.015 | -0.13 |
|  | *Firmicutes* | *Sporobacter* | **0.04** | 0.01 | **0.00** | 0.00 | 0.009 | 0.015 | -0.04 |
|  | *Bacteroidetes* | Unclassified *Rikenellaceae* | **0.03** | 0.01 | **0.00** | 0.00 | 0.009 | 0.015 | -0.03 |
|  | *Deferribacteres* | *Mucispirillum* | **0.16** | 0.07 | **0.00** | 0.00 | 0.009 | 0.015 | -0.16 |
|  | *Firmicutes* | *Coprobacillus* | **0.15** | 0.09 | **0.00** | 0.00 | 0.009 | 0.015 | -0.15 |
|  | *Verrucomicrobia* | *Akkermansia* | **3.06** | 1.20 | **0.00** | 0.00 | 0.009 | 0.015 | -3.06 |
|  | *Firmicutes* | *Blautia* | **0.33** | 0.31 | **0.00** | 0.00 | 0.013 | 0.020 | -0.33 |
|  | *Firmicutes* | Unclassified *Erysipelotrichaceae* | **0.09** | 0.03 | **0.00** | 0.00 | 0.013 | 0.020 | -0.09 |
|  | *Firmicutes* | Unclassified *Firmicutes* | **0.61** | 0.10 | **0.25** | 0.06 | 0.014 | 0.020 | -0.36 |
|  | *Firmicutes* | *Robinsoniella* | **0.50** | 0.38 | **0.00** | 0.00 | 0.014 | 0.021 | -0.50 |
|  | *Firmicutes* | *Gemella* | **0.03** | 0.01 | **0.00** | 0.00 | 0.022 | 0.027 | -0.03 |
|  | *Firmicutes* | *Isobaculum* | **0.00** | 0.00 | **0.01** | 0.00 | 0.022 | 0.027 | 0.01 |
|  | *Firmicutes* | *Marvinbryantia* | **0.06** | 0.02 | **0.00** | 0.00 | 0.022 | 0.027 | -0.06 |
|  | *Firmicutes* | *Acetivibrio* | **0.03** | 0.01 | **0.00** | 0.00 | 0.022 | 0.027 | -0.03 |
|  | *Proteobacteria* | *Parasutterella* | **0.02** | 0.01 | **0.00** | 0.00 | 0.022 | 0.027 | -0.02 |
|  | *Proteobacteria* | *Burkholderia* | **0.00** | 0.00 | **0.69** | 0.66 | 0.022 | 0.027 | 0.69 |
|  | *Proteobacteria* | Unclassified *Burkholderiaceae* | **0.00** | 0.00 | **0.15** | 0.14 | 0.022 | 0.027 | 0.15 |
|  | *Proteobacteria* | *Trabulsiella* | **0.00** | 0.00 | **0.04** | 0.01 | 0.022 | 0.027 | 0.04 |
|  | *Tenericutes* | Unclassified *Mycoplasmataceae* | **0.00** | 0.00 | **0.06** | 0.03 | 0.022 | 0.027 | 0.06 |
|  | *Firmicutes* | *Johnsonella* | **0.04** | 0.01 | **0.00** | 0.00 | 0.036 | 0.041 | -0.04 |
|  | *Proteobacteria* | Unclassified Helicobacteraceae | **0.03** | 0.01 | **0.00** | 0.00 | 0.036 | 0.041 | -0.03 |
|  | *Proteobacteria* | Klebsiella | **0.00** | 0.00 | **0.04** | 0.01 | 0.036 | 0.041 | 0.04 |
|  | *Tenericutes* | Anaeroplasma | **0.01** | 0.01 | **0.00** | 0.00 | 0.036 | 0.041 | -0.01 |
| Group 6 (Low-IgA milk) | *Actinobacteria* | Unclassified *Coriobacteriaceae* | **0.12** | 0.02 | **0.01** | 0.01 | 0.002 | 0.006 | -0.11 |
|  | *Bacteroidetes* | *Odoribacter* | **0.91** | 0.19 | **0.00** | 0.00 | 0.002 | 0.006 | -0.91 |
|  | *Bacteroidetes* | *Parabacteroides* | **1.17** | 0.34 | **0.08** | 0.08 | 0.002 | 0.006 | -1.09 |
|  | *Bacteroidetes* | *Prevotella* | **1.33** | 0.29 | **0.22** | 0.22 | 0.002 | 0.006 | -1.11 |
|  | *Bacteroidetes* | *Rikenella* | **0.40** | 0.12 | **0.00** | 0.00 | 0.002 | 0.006 | -0.40 |
|  | *Firmicutes* | *Dorea* | **0.64** | 0.15 | **0.03** | 0.02 | 0.002 | 0.006 | -0.61 |
|  | *Firmicutes* | *Marvinbryantia* | **0.11** | 0.04 | **0.00** | 0.00 | 0.002 | 0.006 | -0.11 |
|  | *Firmicutes* | Unclassified *Lachnospiraceae* | **15.35** | 3.37 | **2.08** | 1.15 | 0.002 | 0.006 | -13.28 |
|  | *Firmicutes* | Unclassified *Clostridiales* | **8.12** | 1.63 | **2.54** | 1.52 | 0.002 | 0.006 | -5.57 |
|  | *Firmicutes* | *Butyricicoccus* | **0.22** | 0.06 | **0.00** | 0.00 | 0.002 | 0.006 | -0.21 |
|  | *Firmicutes* | *Oscillibacter* | **0.56** | 0.14 | **0.01** | 0.01 | 0.002 | 0.006 | -0.55 |
|  | *Firmicutes* | Unclassified *Ruminococcaceae* | **3.11** | 0.49 | **0.03** | 0.03 | 0.002 | 0.006 | -3.07 |
|  | *Firmicutes* | *Papillibacter* | **0.14** | 0.03 | **0.00** | 0.00 | 0.002 | 0.006 | -0.13 |
|  | *Firmicutes* | *Sporobacter* | **0.06** | 0.01 | **0.00** | 0.00 | 0.002 | 0.006 | -0.06 |
|  | *Firmicutes* | Unclassified *Clostridia* | **0.05** | 0.01 | **0.00** | 0.00 | 0.002 | 0.006 | -0.05 |
|  | *Firmicutes* | *Holdemania* | **0.04** | 0.02 | **0.00** | 0.00 | 0.002 | 0.006 | -0.04 |
|  | *Proteobacteria* | *Desulfovibrio* | **0.12** | 0.03 | **0.00** | 0.00 | 0.002 | 0.006 | -0.12 |
|  | *Proteobacteria* | Unclassified *Desulfovibrionaceae* | **0.05** | 0.01 | **0.00** | 0.00 | 0.002 | 0.006 | -0.05 |
|  | *Proteobacteria* | Unclassified *Desulfovibrionales* | **0.19** | 0.03 | **0.00** | 0.00 | 0.002 | 0.006 | -0.19 |
|  | *Proteobacteria* | *Serratia* | **0.00** | 0.00 | **6.34** | 4.06 | 0.002 | 0.006 | 6.34 |
|  | *Tenericutes* | *Mycoplasma* | **0.04** | 0.03 | **1.12** | 0.32 | 0.002 | 0.006 | 1.09 |
|  | *Actinobacteria* | *Enterorhabdus* | **0.06** | 0.01 | **0.01** | 0.01 | 0.004 | 0.010 | -0.05 |
|  | *Bacteroidetes* | Unclassified *Porphyromonadaceae* | **31.90** | 3.32 | **2.81** | 2.81 | 0.004 | 0.010 | -29.08 |
|  | *Bacteroidetes* | Unclassified *Bacteroidetes* | **1.84** | 0.31 | **0.23** | 0.22 | 0.004 | 0.010 | -1.61 |
|  | *Firmicutes* | *Enterococcus* | **0.05** | 0.01 | **9.18** | 2.75 | 0.004 | 0.010 | 9.13 |
|  | *Proteobacteria* | *Escherichia/Shigella* | **0.00** | 0.00 | **9.09** | 4.15 | 0.004 | 0.010 | 9.09 |
|  | *Deferribacteres* | *Mucispirillum* | **0.13** | 0.08 | **0.00** | 0.00 | 0.009 | 0.017 | -0.13 |
|  | *Firmicutes* | Unclassified *Carnobacteriaceae* | **0.00** | 0.00 | **0.16** | 0.04 | 0.009 | 0.017 | 0.16 |
|  | *Firmicutes* | Unclassified *Enterococcaceae* | **0.00** | 0.00 | **1.50** | 0.41 | 0.009 | 0.017 | 1.50 |
|  | *Firmicutes* | *Coprococcus* | **0.02** | 0.01 | **0.00** | 0.00 | 0.009 | 0.017 | -0.02 |
|  | *Firmicutes* | *Ruminococcus* | **0.11** | 0.06 | **0.00** | 0.00 | 0.009 | 0.017 | -0.11 |
|  | *Proteobacteria* | *Helicobacter* | **0.25** | 0.11 | **0.00** | 0.00 | 0.009 | 0.017 | -0.25 |
|  | *Proteobacteria* | *Enterobacter* | **0.00** | 0.00 | **0.74** | 0.30 | 0.009 | 0.017 | 0.74 |
|  | *Proteobacteria* | Unclassified *Enterobacteriaceae* | **0.00** | 0.00 | **25.36** | 8.09 | 0.009 | 0.017 | 25.36 |
|  | *Proteobacteria* | *Raoultella* | **0.00** | 0.00 | **3.81** | 2.32 | 0.009 | 0.017 | 3.81 |
|  | *Proteobacteria* | Unclassified *Gammaproteobacteria* | **0.00** | 0.00 | **0.16** | 0.05 | 0.009 | 0.017 | 0.16 |
|  | *Bacteroidetes* | *Barnesiella* | **4.99** | 0.59 | **0.90** | 0.89 | 0.010 | 0.017 | -4.09 |
|  | *Bacteroidetes* | *Alistipes* | **1.32** | 0.20 | **0.26** | 0.26 | 0.010 | 0.017 | -1.06 |
|  | *Firmicutes* | Unclassified *Lactobacillales* | **0.51** | 0.14 | **11.83** | 2.74 | 0.010 | 0.017 | 11.32 |
|  | *Firmicutes* | Unclassified *Bacilli* | **0.01** | 0.01 | **0.26** | 0.13 | 0.014 | 0.023 | 0.24 |
|  | *Proteobacteria* | *Pragia* | **0.00** | 0.00 | **0.06** | 0.04 | 0.014 | 0.023 | 0.06 |
|  | *Bacteroidetes* | Unclassified *Bacteroidales* | **6.51** | 0.68 | **1.12** | 1.12 | 0.020 | 0.031 | -5.39 |
|  | *Firmicutes* | *Gemella* | **0.02** | 0.01 | **0.00** | 0.00 | 0.022 | 0.032 | -0.02 |
|  | *Firmicutes* | *Isobaculum* | **0.00** | 0.00 | **0.02** | 0.01 | 0.022 | 0.032 | 0.02 |
|  | *Firmicutes* | *Anaerovorax* | **0.01** | 0.00 | **0.00** | 0.00 | 0.022 | 0.032 | -0.01 |
|  | *Tenericutes* | Unclassified *Mycoplasmataceae* | **0.00** | 0.00 | **0.03** | 0.01 | 0.022 | 0.032 | 0.03 |
|  | *Bacteroidetes* | *Tannerella* | **0.01** | 0.00 | **0.00** | 0.00 | 0.035 | 0.047 | -0.01 |
|  | *Firmicutes* | *Clostridium* | **0.00** | 0.00 | **0.26** | 0.21 | 0.036 | 0.047 | 0.26 |
|  | *Firmicutes* | *Johnsonella* | **0.03** | 0.01 | **0.00** | 0.00 | 0.036 | 0.047 | -0.03 |
|  | *Proteobacteria* | *Parasutterella* | **0.02** | 0.01 | **0.00** | 0.00 | 0.036 | 0.047 | -0.02 |
|  | *Proteobacteria* | *Trabulsiella* | **0.00** | 0.00 | **0.04** | 0.02 | 0.036 | 0.047 | 0.04 |

* Difference in the mean value for Pre and Day 14 samples
